# The path to international medals: A supervised machine learning approach to explore the impact of coach-led sport-specific and non-specific practice

**DOI:** 10.1371/journal.pone.0239378

**Published:** 2020-09-25

**Authors:** Michael Barth, Arne Güllich, Christian Raschner, Eike Emrich

**Affiliations:** 1 Department of Sports Science, Saarland University, Saarbrücken, Germany; 2 University of Applied Sciences Kufstein Tirol—FH Kufstein, Kufstein, Austria; 3 Department of Sport Science, University of Kaiserslautern, Kaiserslautern, Germany; Gachon University Gil Medical Center, REPUBLIC OF KOREA

## Abstract

Research investigating the nature and scope of developmental participation patterns leading to international senior-level success is mainly explorative up to date. One of the criticisms of earlier research was its typical multiple testing for many individual participation variables using bivariate, linear analyses. Here, we applied state-of-the-art supervised machine learning to investigate potential non-linear and multivariate effects of coach-led practice in the athlete’s respective main sport and in other sports on the achievement of international medals. Participants were matched pairs (sport, sex, age) of adult international medallists and non-medallists (n = 166). Comparison of several non-ensemble and tree-based ensemble binary classification algorithms identified “eXtreme gradient boosting” as the best-performing algorithm for our classification problem. The model showed fair discrimination power between the international medallists and non-medallists. The results indicate that coach-led other-sports practice until age 14 years was the most important feature. Furthermore, both main-sport and other-sports practice were non-linearly related to international success. The amount of main-sport practice displayed a parabolic pattern while the amount of other-sports practice displayed a saturation pattern. The findings question excess involvement in specialised coach-led main-sport practice at an early age and call for childhood/adolescent engagement in coach-led practice in various sports. In data analyses, combining traditional statistics with advanced supervised machine learning may improve both testing of the robustness of findings and new discovery of patterns among multivariate relationships of variables, and thereby of new hypotheses.

## Introduction

The pursuit of international success as well as its public funding and private sponsorship presuppose knowledge of effective means and thus of the fundamentals of the *production function* of sporting success. We must understand *which features* predict the development of exceptional international success and in *which way* they do.

A review of the relevant literature revealed several theoretical, descriptive, and normative athlete development frameworks; notably, the Stages of Talent Development [1], the Deliberate Practice concept (DP) [2], the Developmental Model of Sport Participation (DMSP) [3], the Long Term Athlete Development [4], the Differentiated Model of Giftedness and Talent [5], the Life-span Model of the Acquisition and Retention of Expert Perceptual-motor Performance [6], the Athletic Talent Development Environment Model [7], the Three Dimensional Athlete Development Model (3D-AD) [8], and the Foundations, Talent, Elite and Mastery (FTEM) Framework [8, 9]. The number of athlete development frameworks is growing and they are becoming more complex, postulating complex interactions of personal (genetic endowment, psychological skills, personality traits) and environmental factors (practice, opportunities, social support, lifestyle, athlete support programs). *Practice* during childhood and adolescence is conceptualised as one of the central manipulable factors in athlete development models. Other personal and environmental factors are largely regarded in an *instrumental* role to long-term extensive practice.

Besides coach-led practice, two frameworks [1, 3] proposed beneficial effects of “deliberate play” i.e. informal childhood/adolescent peer-led sports play without coach supervision. However, a recent systematic review of empirical studies did not provide support for this latter hypothesis [10]. In the present study, we focus on *coach-led practice* in organised settings (e.g., sport clubs, high school sport, sport academies).

A recent article [11] reviewed studies addressing the relevance of coach-led practice for international success in the highest, open-age category (i.e., senior success). Table 1 summarises available empirical results regarding effects of the volume of coach-led practice in the athlete’s respective main sport (henceforth: main-sport practice) and in other sports (henceforth: other-sports practice).

**Table 1:** Relevance of the volume of coach-led practice in an athlete’s main sport and in other sports for the differentiation between senior international and national-level success (adapted and updated from [11]).

		Amount of main-sport practice		Amount of other-sports practice	
No	Source	Childhood and adolescence	Adulthood	Childhood and adolescence	Adulthood
1	[12]	–	n.a.	+	n.a.
2	[13]	+/o	n.a.	n.a.	n.a.
3	[14]	o	n.a.	+/o	n.a.
4	[15]	o/–	o	o	o
5	[16]	o/–	o	+	+
6	[17]	o	o//–	o/+	o//+
7	[18]	–	o/n.a.	+	o/n.a.
8	[19]	o	o	+	n.a./o
9	[20]	o//–	o	o//+	o

*Note*: n.a.: no information available

Relevance for success:

+: sig. positive correlation (athletes achieving international senior-level success practiced more compared to ‘only’ nationally successful athletes)

–: sig. negative correlation (internationally successful athletes practiced less compared to ‘only’ nationally successful athletes)

o: no correlation between success and amount of practice

x/y: the majority of the results in this category correspond to x, but y was also found

x//y: x and y were found the same number of times

Note that study 2 [13] compared more successful *Greek* with less successful *Canadian* gymnasts.

Regarding the effect of childhood/adolescent main-sport practice on senior international success, the results have been inconsistent. While some studies found no or negative effects, one study that involved rhythmic gymnasts (mean age 17.3 years) [13] reported in parts greater amounts of gymnastics practice among six more successful Greek than six less successful Canadian gymnasts. Interestingly, several studies found a positive effect of childhood/adolescent coach-led other-sports practice on senior international success (Table 1)—an observation that is not adequately explained by the tenets of either of the two most established concepts of talent development, the deliberate practice view and the DMSP, nor by the other concepts mentioned above.

Although research into athlete development has been extensive, it is still mainly explorative. Current theoretical concepts of talent development imply non-linear, multivariate effects of multiple predictors; by contrast, extant research has typically only involved traditional bivariate analyses using linear methods (i.e., presupposing an appropriate data model and estimating parameters for this model based on the data) and was perhaps not fully appropriate.

To avoid proceeding from a data model and, instead, use general-purpose learning algorithms to *learn* about the relationship between the response and its predictive features [21], advanced state-of-the-art *supervised machine learning* may be appropriate. Furthermore, these procedures allow controlling for confounding variables, an issue that has scarcely been adequately considered (cf. [11]). Supervised machine learning has very recently been introduced into the investigation of athletes’ developmental participation patterns using Gradient Boosting Machine (GBM) (basically [22]; application [23]) and an advancement of GBM called “eXtreme gradient boosting” (XGBoost) [11, 24].

The present study addressed the issues of earlier research discussed above in two ways. First, it applied supervised machine learning and thereby extended earlier research by investigating potential *non-linear* effects of main-sport practice and other-sports practice on international success and also potential *interactions* between main-sport and other-sports practice. Second, it examined a data set of matched pairs of international medallists and non-medallists matched on type of sport, sex and age of athletes’ present career peak performance, thereby controlling for potential confounds of these variables (cf. [11]). The data set was first presented by [18], then only conducting traditional group comparison statistics.

## Methods

We differentiated between international medallists and non-medallists in a sample of German senior national squad athletes. Thus, our task can be described as a binary classification problem. The subsample of medallists included 38 Olympic and World Champions, 27 Olympic/World Championship silver/bronze medallists and 18 European Champions. A matching procedure within a representative sample of national squad athletes from all Olympic sports [16] assigned a matched non-medallist to each medallist. The matching was based on type of sport, sex, and age of career peak performance [18]. The non-medallists were senior national squad members but never won an international senior-level medal. The procedure resulted in a sample of 83 pairs of international medallists and non-medallists (n = 166; 86 males, 80 females). The original study received ethical approval from the German Federal Institute of Sports Science (BISp). The ethics committee of the Saarland University confirmed that no repeated ethical approval was necessary for the re-analysis of the existing and approved data.

To classify the groups, we used athletes’ cumulative hours of coach-led main-sport and coach-led other-sports practice through three age categories: until 14, 15 to 18 and 19 to 21 years. They are described in Table 2. The missing values at 19 to 21 years were due to two athlete pairs aged below 19 years. Since the values were not missing at random we decided to exclude the two athlete pairs from further analysis.

**Table 2:** Senior international medallists’ and non-medallists’ main-sport and other-sports coach-led practice hours through three age categories: Until 14, 15 to 18 and 19 to 21 years.

	Medallists			Non-Medallists		
	M	(SD)	n	M	(SD)	n
Age (years)	25.0	4.7	83	24.2	4.4	83
Age of peak performance (years)	23.5	3.9	83	23.3	4.0	83
Main-sport practice (hours)						
until 14 years	1005.7	1057.0	83	1482.4	1246.0	83
15 to 18 years	2300.3	1451.2	83	2783.0	1648.8	83
19 to 21 years	2783.7	1503.2	81	3007.0	1422.5	81
Other-sports practice (hours)						
until 14 years	728.6	1021.8	83	179.7	336.0	83
15 to 18 years	274.7	505.3	83	60.5	133.1	83
19 to 21 years	90.0	245.9	81	63.5	191.5	81

The data analyses proceeded in four major, progressive steps: 1. To compare different procedures to identify the best-performing algorithm; 2. to determine the importance of each feature; 3. to analyse “individual conditional expectation” (ICE) curves—an analysis providing expected classifications for every instance of each feature; and 4. to examine the interaction of the two most important features.

In advance of applying a certain algorithm to our classification problem, we compared the performance of one non-ensemble method (Support Vector Machines (SVM)) and two tree-based ensemble binary classification algorithms (Random Forests (RF) and XGBoost; for their basic description see e.g. [25]). We built and tuned our models using the caret package (v6.0–84; function xgbTree for XGBoost and rf for RF; [26]). Optimizing of tuning parameters was implemented using “tuneLength”, a built-in model within caret to generate random tuning parameter combinations [26]. Given the limited sample size, resampling and model evaluation were done by leave-one-out cross validation. This means that each fold (outer loop) was trained by application of different tuning parameters (inner loop). The procedures yielded a total of 81,000 predictions. The final parameters were determined by choosing the model with the highest value of the area under the receiver operating characteristic curves (AUC).

AUC was also used to assess and compare the discrimination performance of our optimised SVM, RF and XGBoost models. An AUC of 1 indicates a perfect model, a value of 0.5 represents the performance of a random classifier that does not have any discriminative power [25, 27]). Roughly classified, the AUC can be interpreted as follows: excellent (1.00 ≤ AUC ≥ 0.90), good (0.90 < AUC ≥ 0.80), fair (0.80 < AUC ≥ 0.70), poor (0.70 < AUC ≥ 0.60), and failed (0.60 < AUC ≥ 0.50) (basically, [28]).

The respective results (AUC, Precision, Recall, f1) for the optimised models are shown in Table 3.

**Table 3:** Assessed classification accuracy of different methods.

Indicator	Support Vector Machines	Random Forests	eXtreme Gradient Boosting
AUC	0.71	0.78	0.79
Precision medallists	0.72	0.70	0.71
non-medallists	0.67	0.71	0.73
Recall medallists	0.63	0.72	0.74
non-medallists	0.75	0.69	0.69
f1 medallists	0.67	0.71	0.72
non-medallists	0.71	0.70	0.71

Baseline values are in all cases 0.50.

Not only relative to the results, but also to address—at least to some further extent—the potential issue of overfitting with XGBoost, we decided to do all further analysis by application of the mentioned algorithm. Tuning parameters for our final XGBoost model were: nrounds = 50, max_depth = 5, eta = 0.3, gamma = 0, colsample_bytree = 0.8, min_child_weight = 1, subsample = 0.625. Max_depth is the maximum depth of a leaf node to the root of the tree (default value = 6). These hyperparameters “are primarily used to balance overfitting with the accuracy and computational complexity” [25]. We reduced the value to two to directly control model complexity (we forewent reduction to one, which would generate decision stumps). Furthermore, we reduced eta (default value = 0.3) to 0.1 to make training more robust to noise and therefore increased nrounds to 150 [29]. The resulting AUC of 0.75 is comparable to the one of our final model.

Since XGboost and the algorithms are new developments that have only been applied a few times yet and because we aimed to perform a robustness-check, we decided to use two functions to calculate the feature importance: first, the “xgb.importance” function implemented in the “xgboost” package and second, the “FeatureImp” function, which is part of the “iml” package.

In the “xgb.importance” function, the higher the value (“gain”), the more important the feature for the model [29]. The feature importance in the XGBoost package is similar to the one in the “gbm” package (function: “rel.inf”), which is based on the formulae developed by Friedman [22]. “The measures are based on the number of times a variable is selected for splitting, weighted by the squared improvement to the model as a result of each split, and averaged over all trees” [21]. Each variable’s relative contribution is scaled with their sum adding to 100 [21].

In the second, “FeatureImp” function, a feature’s importance is measured by the increase of the model’s prediction error after permuting the feature. If the feature’s value is permuted and the model’s error remains unchanged, the feature is “unimportant” to the model [30]. Based on this, the ratio between “permutation error” and “original error” was calculated, which means features with an importance of (around) 1 are not relevant for the model.

Since feature importance does not say anything about the *direction* and the *way* in which a feature influences the response, the “individual conditional expectation (ICE) curves” [31] are presented for each feature. Implementation was done using the “pdp” package version 0.6.0 [31]. No trimming of outliers was done.

ICE curves visualize local expectations—i.e. expectations for every instance of a feature. When averaging the ICE curves (here: arithmetic mean) we get a so called “partial dependence plot” (pdp) for the feature. The partial dependence plot shows how the prediction in a data set changes on average (over all instances) when a feature is changed and simultaneously all other features are held “constant”. It should be noted that assumingly, the feature for which the partial dependence plot is computed is distributed independently from the other features of the model [30]. This assumption may not be unproblematic. Therefore, possible interactions between the predictive features should be examined.

We investigated interactions between the model’s most important features, performing the calculation of interactions’ importance by the “Interaction” function (package: “iml”, version: 0.9.0; cf. [32]). This interaction was then examined using a three-dimensional partial dependency plot (“pdp” package).

Implementations of all procedures were done in the R environment (v3.6.1).

## Results

**Fig 1:**
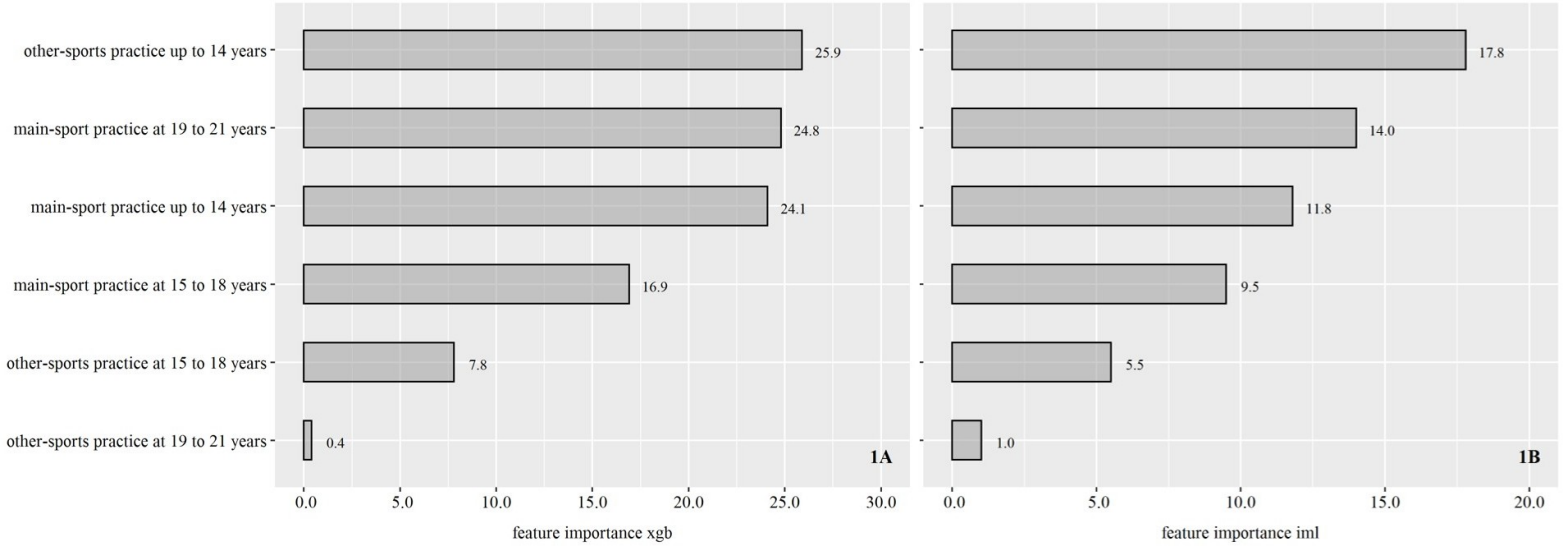
Importance of the model’s features. *Note*: A: Each variable’s relative contribution is scaled with the sum of all adding to 100. B: Ratio between “permutation error” and “original error” was calculated, which means features with an importance of (around) 1 are not relevant for the model.

The mean AUC was 0.79. That is, the model showed *fair* (nearly good) discrimination power. Fig 1 presents the importance of the model’s features.

The two variants of calculating feature importance showed identical results with regard to the order of features’ importance. Coach-led “other-sports practice until age 14 years” was the discriminating feature with the highest importance (see Fig 1). However, when using “xgb.importance”, other-sports practice until age 14 years, main-sport practice at 19 to 21 years and main-sport practice until 14 years showed comparable values. Other-sports practice at 19 to 21 years had no relevance to athletes’ classification, irrespective of the way of calculation.

**Fig 2:**
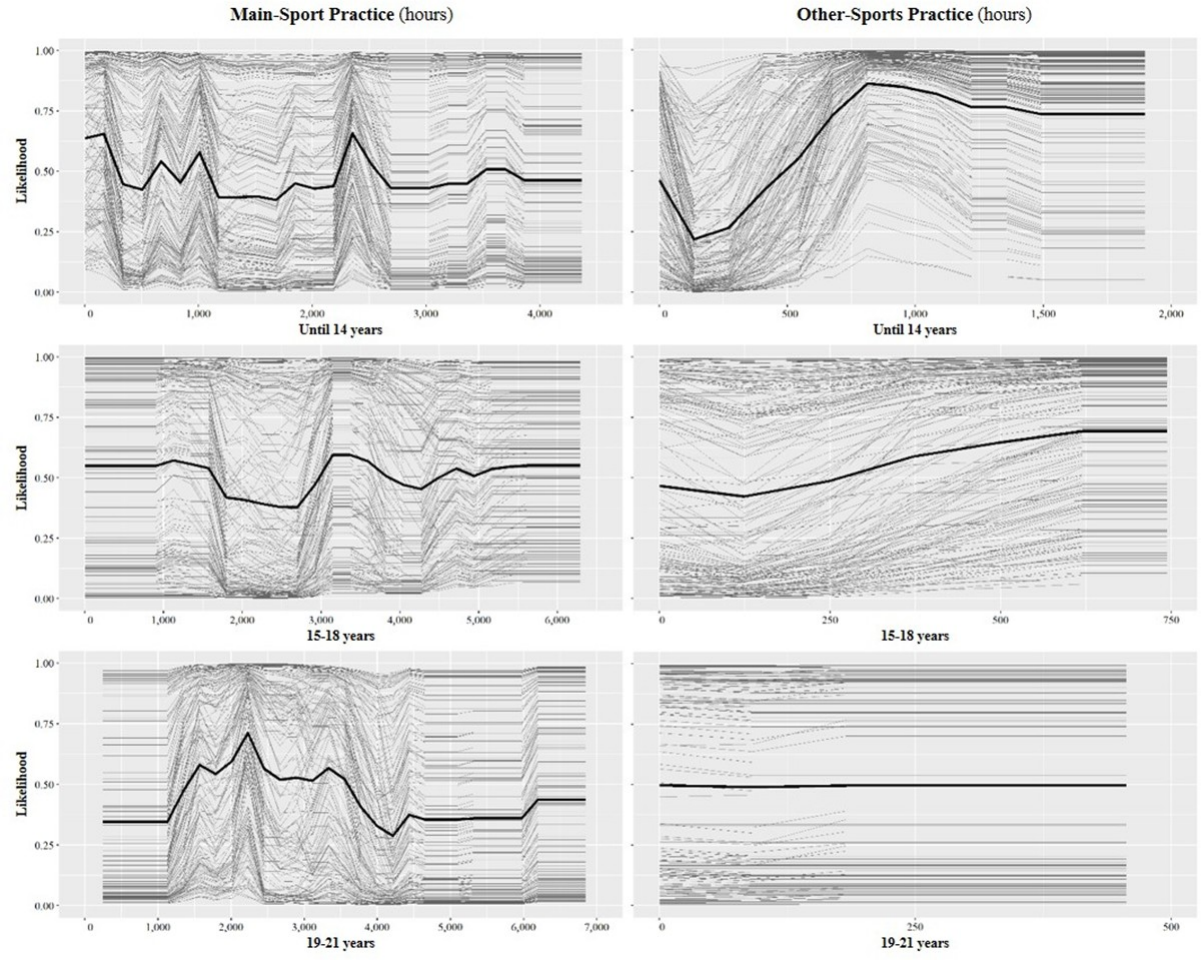
Individual Conditional Expectation (ICE) curves for the features volume of coach-led practice in the athlete’s main sport and coach-led practice in other sports. Bold curves illustrate the mean values. A likelihood value of 0.5 corresponds to chance. Note the different abscissae scale orientations.

The ICE curves for all model features are shown in Fig 2. The discriminating effects of the two most important features, other-sports practice until age 14 years and main-sport practice at 19–21 years, clearly display *non-linear* patterns. Other-sports practice until age 14 years shows a *saturation* pattern (see Fig 2, top right): Amounts up to ~300 hours were associated with a probability below chance to become an international medallist. With increasing amount of other-sports practice, the probability to win international medals increased dramatically up to a value of 0.86 at ~800 accumulated hours. Further expansion of other-sports practice did not further increase the chance to become a medallist.

Main-sport practice at 19–21 years displayed a *parabolic* pattern (see Fig 2, bottom left): The probability to become a medallist was greatest (0.72) when accumulating ~2,200 hours (corresponding to ~14.7 hours/week) and was below chance when accumulating either less than ~1,200 or more than ~4,000 hours. Interestingly, the curves for main-sport practice until age 14 years and at 15 to 18 years oscillate around 0.5, i.e. chance (Fig 2, top left and centre left).

**Fig 3:**
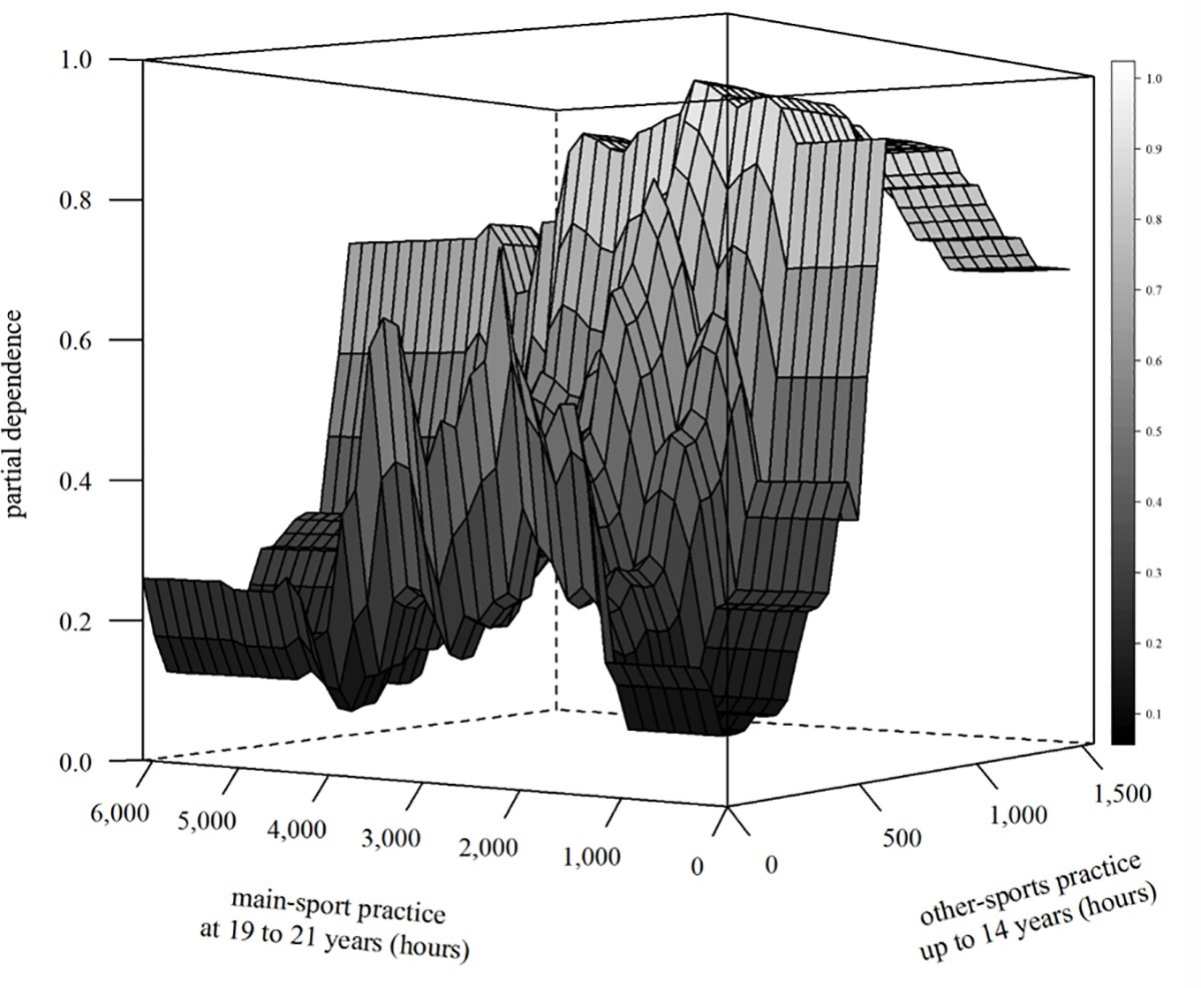
Interactive effect of the features coach-led other-sports practice up to 14 years and coach-led main-sport practice at 19 to 21 years on the probability to be classified as an international medallist. A partial dependence value of 0.5 corresponds to chance. Note the different scale orientations of the abscissae.

The two most important features, coach-led other-sports practice until age 14 years and coach-led main-sport practice at 19–21 years, also displayed the highest two-way interaction strength (0.33). Fig 3 illustrates this (weak) positive interaction. The probability to be an international medallist was increased when less than ~3,600 hours of main-sport practice at 19–21 years were combined with ~700–1,150 hours of other-sports practice until age 14 years. This chance was the highest, exceeding 0.95, when combining ~1,400–2,100 main-sport practice hours at 19–21 years (~9.3–14.0 hours/week) with ~730–1,020 other-sports practice hours accumulated until 14 years of age. On the other hand, the probability to win international medals was reduced when either combining less than ~1,200 hours of main-sport practice at 19–21 years with less than ~500 hours of other-sports practice until age 14 years or combining more than ~3,700 main-sport practice hours at 19–21 years with less than ~700 other-sports practice hours until 14 years of age.

## Discussion

The study used a state-of-the-art approach from supervised machine learning, XGBoost, to analyse developmental participation data of international medallists and non-medallists. Overall, the model had fair (nearly good) discrimination power.

The first finding was that coach-led other-sports practice until age 14 years stood out as the model’s most important feature, while the probability to be an international medallist was widely unrelated to the amounts of coach-led main-sport practice until age 14 and 18 years. Perhaps even more significantly, the present analyses extend earlier findings by revealing *non-linear* relationships of international medalling success with the most important features. Childhood/adolescent other-sports practice displayed a *saturation* pattern: Low amount (< ~300 hours) was associated with reduced medalling probability; this probability increased (up to 0.86) with growing other-sports practice up to ~800 hours, but further expansion did not further increase the likelihood to win international medals. It should be noted, however, that only 18.8% of athletes engaged in more than these 800 hours.

Main-sport practice at 19–21 years displayed a *parabolic* pattern with the peak probability to be a medallist around 2,200 hours. Moreover, interaction analyses indicated that the probability to be an international medallist was the highest (> 0.95) when combining ~1,400–2,100 hours of main-sport practice at 19–21 years (~9.3–14.0 hours/week) with ~730–1,020 hours of other-sports practice accumulated until age 14 years. Medalling probability was below chance when combining little childhood/adolescent other-sports practice with either very little (< 1,200 hours) or very extensive (> 3,700 hours) main-sport practice at 19–21 years.

Interestingly, the majority of the athletes (64%) invested amounts of main-sport practice that were above the optimal range as per the present analysis—consistent with a small negative effect of the volume of main-sport practice noted in Güllich’s [18] group comparison (see also Table 2 above). The athletes’ absolute volume of other-sports practice was much smaller than of main-sport practice (see Table 2), but the former differentiated international medallists from non-medallists to a much greater extent. The beneficial effect of childhood/adolescent coach-led other-sports practice on adult world-class success clearly speaks against some of the central assumptions of the deliberate practice view [2] and the “elite performance through early specialization” pathway of the DMSP [3], which suggested focusing on intensified specific practice in a single sport. Instead, among senior high performers similarly engaging in multi-year sport-specific practice, a broadened variability of childhood/adolescent participation in multi-sport practice apparently increases the probability of the emergence of adult exceptional performers (for hypotheses on potential mechanisms, see [16, 18]).

Strengths of the present study included the application of state-of-the-art supervised machine learning procedures involving investigation of non-linear and multivariate effects in a considerable, matched-pairs sample involving the world’s best athletes. But the present study does have limitations. First, the data collection was based on a retrospective survey, which not only implies the common constraints in power but also that the findings are observational and not causal [18]. Second, the present sample was large relative to the population of international medallists, who are scarce by definition, but it was relatively small in absolute terms. In addition, we only included a few variables in the model (those considered to promise greatest leverage). Furthermore, machine learning has hardly ever been applied to athletes’ practice data with respect to the present research question before. We sought to address this weak point by comparing different procedures and also applying leave-one-out cross-validation. In addition, we compared the AUC of various models, systematically manipulating hyperparameters directly controlling model complexity.

Third, the micro-structure and quality of practice were not taken into account. The model did also not consider scholastic physical education because it is highly standardised by obligatory curricula throughout Germany.

Besides, a general issue of empirical research into athletes’ participation patterns has not been considered in the research literature and also applies to the present study. The range of athletes’ empirically implemented practice regimes is societally constricted by sport-specific practice cultures, traditions, sport federations’ practice frameworks and coach education. That is, we can only empirically observe a narrowed sector of conceivable practice activities, where the range is constricted by normative imperatives of the ‘sport system’ (for rationalised myths see generally [33]). This narrowed range may not only affect outcomes, but also constrict investigation of the full range of conceivable participation patterns and compositions of varying types of practice activities.

The results clearly show that adequate data analysis procedures for the present classification problem have to be able to explore potential non-linear and multivariate effects of predictive features.

Appropriate frameworks for future research should envisage the interplay of nature and nurture including the incorporation of further factors, such as genetic endowment and gene-environment interaction [34], other forms of preparation (e.g., psychological preparation; cf. [35]) or contextual factors (e.g., socioeconomic factors [36]). Furthermore, we should also consider “situative factors” (e.g., management of competition anxiety; [11]).

Another valuable approach may be to organise and pragmatically tailor theoretically derived concepts, rather than attempting to include all potential factors [37]. In this, due to the fact that senior international medallists are per se a small population, sport-specific studies—although valuable—are difficult to realise with sufficient power. Thus, new ways to categorise and combine different sports based on various similarities may be promising (e.g. pooling athletes from team game sports, artistic composition sports or typical endurance sports [16]).

Combining traditional statistics with advanced supervised machine learning may improve both testing of the robustness of findings and new discovery of patterns among multivariate relationships between variables. To ensure generalisability, the assessment of models on further test data sets is requested. We therefore see one of the great benefits of the procedure in the development of new hypotheses, which seems to be justified and necessary in an exploratory field of research such as the one at hand.

It can be concluded that the research findings question excessive specialised main-sport practice at an early age and call for engagement in coach-led practice in various sports through childhood/adolescence. Existing institutionalised programmes for talent development—in essence pursuing the expansion of the available sport-specific practice time and more intensive usage of the available time (extensive and intensive time economy; for economics of time in practice see [12])—probably induce excess involvement in childhood/adolescent specialised main-sport practice.

Generally, there is a clear lack of theoretically derived and empirically substantiated frameworks for athlete development, which opens the door for future research in this field.
