# Correction: The path to international medals: A supervised machine learning approach to explore the impact of coach-led sport-specific and non-specific practice

**DOI:** 10.1371/journal.pone.0244509

**Published:** 2020-12-18

**Authors:** Michael Barth, Arne Güllich, Christian Raschner, Eike Emrich

The affiliation for the third author is incorrect. The correct affiliation is not indicated. Christian Raschner is not affiliated with #2 but with: Department of Sport Science, University of Innsbruck, Innsbruck, Austria.
